# Supplementary material for: Are young and older children with diarrhea presenting in the same way?
Source: PLoS One. 2024 May 13;19(5):e0300882. doi: 10.1371/journal.pone.0300882 (PMC11090295; doi:10.1371/journal.pone.0300882)
Supplement: Attachment — Submitted filename: Response letter.docx [file pone.0300882.s002.docx]

**Rebuttal letter**

Date:	January 16, 2024

To: The Editor,

PLOS One

From: Rina Das

Corresponding author,

Manuscript Ref.: MS ID#: [PONE-D-23-25038] - [EMID:f90d745b64f65c11]

Subject: Response to the comments of the reviewers of on MS ID#: [PONE-D-23-25038] - [EMID:f90d745b64f65c11]entitled " **Are young and older children with diarrhea presenting in the same way?** "

Thank you for allowing us to submit our revised manuscript with revision. We have modified the manuscript according to your and the reviewers' suggestions. We are sending a track-change version of the manuscript highlighting our changes for your convenience. We are also attaching this rebuttal letter outlining a point-by-point response to each issue kindly raised by the respected reviewers. We hope our responses will be appropriate to qualify the manuscript for publication in your well-reputed journal. We are expressing our sincere thanks to the reviewers and the academic editor for the overall positive comments.

**Comments to the author:**

In this instance, it seems there may be acceptable restrictions in place that prevent the public sharing of your minimal data. However, in line with our goal of ensuring long-term data availability to all interested researchers, PLOS’ Data Policy states that authors cannot be the sole named individuals responsible for ensuring data access (<http://journals.plos.org/plosone/s/data-availability#loc-acceptable-data-sharing-methods>).
Data requests to a non-author institutional point of contact, such as a data access or ethics committee, helps guarantee long term stability and availability of data. Providing interested researchers with a durable point of contact ensures data will be accessible even if an author changes email addresses, institutions, or becomes unavailable to answer requests. Before we proceed with your manuscript, please also provide non-author contact information (phone/email/hyperlink) for a data access committee, ethics committee, or other institutional body to which data requests may be sent. If no institutional body is available to respond to requests for your minimal data, please consider if there any institutional representatives who did not collaborate in the study, and are not listed as authors on the manuscript, who would be able to hold the data and respond to external requests for data access? If so, please provide their contact information (i.e., email address). Please also provide details on how you will ensure persistent or long-term data storage and availability.

**Response:**

Thank you for your valuable comment. In the methodology section of the manuscript, we added the data availability statement. We also provide non-author contact information from icddr,b Research Administration as “Data Availability: This data set contains some personal information of the study patients (such as name, admission date, month, area of residence). Our IRB has required that the personal information of the participants is not disclosed. Thus, the policy of our centre (icddr,b) is that we should not make the availability of the whole data set in the manuscript, the supplemental files, or a public repository. However, data related to this manuscript are available upon request and researchers who meet the criteria for access to confidential data may contact **Armana Ahmed (armana@icddrb.org)** to the Research Administration of icddr,b (http://www.icddrb.org/).”
