# Supplementary material for: Are young and older children with diarrhea presenting in the same way?
Source: PLoS One. 2024 May 13;19(5):e0300882. doi: 10.1371/journal.pone.0300882 (PMC11090295; doi:10.1371/journal.pone.0300882)
Supplement: Attachment — Submitted filename: Reviewer comments.docx [file pone.0300882.s001.docx]

**Comments**

**Major comments:**

1) Your manuscript requires major revision of % writing
2) First of all, your outcome (dependent variable) should be “diarrhea” for your Table 1 and Table 2. Therefore, you have to re-construct Table 1 and Table 2 using ‘Diarrhea’ as outcome variable and all the variables listed in both tables as independent variables using bivariable logistic regression to calculate the unadjusted odds ratio. Later on, those variables socring *p*-value ≤ 0.25 to take them to the final model (Multivariable logistic regression).
3) Your Table 3 is incorrect. You have to construct another Table 3 which mainly focuses on multivariable logistic regression by taking those variables from Table 1 and Table 2 (after incorporating the comments given in the above 2^nd^ major comments) which scored *p*-values less than or equal to 0.25. Mind you, you have to analyses both unadjusted odds ratio and adjusted odds ratio in Table 3 after including those variables with a score of *p*-value ≤ 0.25. Therefore, all your Tables (Table 1, Table 2, and Table 3) are incorrect. With these incorrect analyses you cannot, discuss, you cannot conclude and you cannot recommend. Therefore, I will stop reviewing your manuscript now. This is because, all your results, discussions, conclusion and recommendations will definitely be changed after complying with the comments given above. Mind you, this is your major of major revisions. Therefore, I will review your second manuscript after incorporating all what has been commented above.

**Minor comments:**

1) Avoid unnecessary open space between title and authors name and between authors name and affiliation. Example, line number 2 and 5 have nothing to indicate
2) Your Table 4 may be correct, but I will see it after you incorporated all the major comments given above.

**Sections**

**Page-2**

**Abstract**

Rewrite your abstract using the structured way of abstract writing i.e. divide it into sub-sections **Background, Method, Results, and Conclusion**

L25: every single digit number should be written in word. Therefore, write <5 in word “less than	five” and do the same correction throughout the document

L26: delete “children” and replace it by “diseases”

L26 – L27: the sentence “More young children are now surviving and improving the survival of older	children” is meaningless. Re-write it meaningfully!!!

L33: Every time the word “respectively” should be preceded by comma. Therefore, add comma	after the word “age”

L38: Don’t start a sentence with abbreviation; rather write in full form as “*Vibrio cholerae*”

**Page-3**

**Introduction**

L75 – L76: The justification “In recent decades, little is known about the burden of diarrheal disease	in older children and adolescents” doesn’t go in line with your title. Therefore, modify it as	“In recent decades, little is known about the burden of diarrheal disease in children under five	and in older children five to nine years”

**Page-4**

L81: add ‘s’ to statement and make it “Statements”

L86: Don’t start a sentence with abbreviation ‘ERC’. Rather, write the full form ‘Ethical Review	Committee’

**Page-5**

L93: Same comment as above, write the full form of DDSS

L104: the 11^th^ reference should be placed between SPSS and the full stop

L108: Delete ‘0-<5’ and replace it by ‘under five’

**Page-6**

L110 – L111: Delete all the (%) from the two lines

**Page-7**

L133: Delete ‘simple’ and replace it by ‘bivariable’

L136: Delete ‘< 0-5 years’ and replace it by ‘less than five years’

L146: Delete colon from ‘Result:’

L147 – L148: the sentence “From 2012 to 2021, in Dhaka Hospital, out of a total of 28,781	surveillance patients, 5 to 9 years of children were 614 (%), randomly selected 2456 (%) children	from 14,684 under -5 children (Figure 1).” is non-sense. Please re-write it meaningfully

L149 – L151: still the sentence “During the study period, in Matlab Hospital, out of 12,499	surveillance patients, 5 to 9 years children were 278 (%), and we randomly selected	1112 (%) children from 6,460 under-five children (Fig 1).” is non-sense. Re-write it	meaningfully

**Page-8**

L160: Don’t start a sentence with number. Write it as “Five to nine years”

L164 – L 166: From Table 1 delete ‘Ref’ and replace it by ‘1’. This is because you are taking	that option as a reference, so it will have an Odds Ratio of 1. Furthermore,	italicize *CI* and *p* from p-value. Similarly, do the same correction throughout the	manuscript.

In this Table 1 please enclose the *CI*s of the variables ‘**The water treatment** **method used**’ and ‘**Toilet facility**’.	Moreover, where are the ORs with the CI for the variables ‘**Presence of convulsion**’ and	‘Outcome’?

From Table 2 for the variable ‘Maternal education’ you have to take the option ‘Literate’ as a	reference; therefore, delete the OR with its *CI* and replace it by 1

**Comments on References**

**Reference #3**: It is too old data in 2008, it doesn’t describe the current situation. Therefore,	remove it and replace it by the most recent one

**Reference #4**: It is too old data in 2008, it doesn’t describe the current situation. Therefore,	remove it and replace it by the most recent one

**Reference #9**: Lacks page number. Therefore, include the page number after going back to the	original article

I will continue evaluating the references in your second draft after addressing the major comments given above!!!
